# Spina bifida, diplomyelia, and Chiari-like malformation in an Aberdeen Angus calf

**DOI:** 10.1186/s12917-025-05108-w

**Published:** 2025-10-28

**Authors:** Anika Korte, Enrice Hünerfauth, Sina Reinartz-Albrecht, Katarzyna Marek, Andrea Tipold, Ottmar Distl, Marion Hewicker-Trautwein, Maike Heppelmann

**Affiliations:** 1 Clinic for Cattle, University of Veterinary Medicine, Foundation, Hannover, Bischofsholer Damm 15, Hannover, 30173 Germany; 2 Clinic for Small Animals, University of Veterinary Medicine, Hannover, Germany; 3 Institute of Animal Genomics, University of Veterinary Medicine, Hannover, Germany; 4 Department of Pathology, University of Veterinary Medicine, Hannover, Germany

**Keywords:** Calf, Congenital malformations, MRI, Gene GRM7

## Abstract

**Background:**

Spina bifida and Chiari-like malformation are rare congenital anomalies resulting in severe neurological signs in calves. The cause is unclear, but genetic and environmental factors are thought to play a role.

**Case presentation:**

A one-day-old, female, Aberdeen Angus calf was referred to the Clinic for Cattle, University of Veterinary Medicine, Hannover, Germany, because of inability to stand. The calf had a circular hairless area located over the caudal part of the sacrum and showed paraparesis, reduced spinal reflex responses in the hind limbs, ventral strabismus, and exophthalmos. Intracranial and hind limb lower motor lesions were suspected. Radiography and magnetic resonance imaging showed a congenital absence of the sacral vertebral arch from sacral vertebra (S) 3 to S5, a hydro-meningomyelocele, deformation of the four brain ventricles, and severe herniation of the cerebellum through the foramen magnum. The calf was humanely euthanised, and a postmortem examination showed severe congestion and herniation of the cerebellar vermis. The vertebral arches of S3, 4, and 5 were incompletely developed to absent, and the cervical spinal cord had dilatation of the central canal (hydromyelia) and bilateral cavitation (syringomyelia). Additional central canals with a smaller diameter (diplomyelia) were also present. Whole genome sequencing revealed *GRM7* as a possible candidate gene associated with cerebellar displacement and Chiari-like malformation.

**Conclusions:**

This case report highlights the importance of including congenital malformations in the differential diagnosis of calves with neurological signs. Such abnormalities are often challenging to diagnose and require imaging procedures. Further research is needed to determine the genetic basis of these malformations.

**Supplementary Information:**

The online version contains supplementary material available at 10.1186/s12917-025-05108-w.

## Background

Spina bifida and Chiari-like malformation are rare congenital defects in calves, frequently occurring together [1–6]. Partial failure of dorsal neural tube closure during embryogenesis results in the absence of the dorsal parts of the vertebrae, and the spinal arch remains open dorsally [7]. Spina bifida has various forms and locations in humans, and although the entire spinal cord may be affected, only the lumbar or sacral areas are usually involved. Spina bifida occulta is characterised by a gap in the dorsal arch of affected vertebrae, with the spinal cord and meninges located within the spinal canal. This form in humans may be symptomless when only individual vertebrae are affected [8]. In spina bifida cystica, a cystic bulge develops above the affected vertebrae. When only parts of the neural membranes protrude, it is defined as a meningocele. The term myelomeningocele is used when parts of the spinal cord are involved in the herniation.

In cattle, spina bifida occurs mainly in the lumbosacral area [9]. Calves with spina bifida often do not undergo a veterinary examination, or the defect is not documented; thus, the frequency and types of this malformation are unknown. Spina bifida occulta is characterised by a hairless fluctuant area in the dorsal midline of the spine [3]. In cattle, it is often associated with Chiari-like malformation, a complex developmental abnormality, with protrusion of parts of the cerebellum through the foramen magnum into the cervical spinal canal [10]. This may be associated with syringomyelia, also reported in dogs [11, 12]. In humans, five different types of Chiari malformation are distinguished and classified according to the structures affected and whether posterior cranial fossa deformation is present. Common other signs alongside with Chiari malformation in humans are syringomyelia, internal hydrocephalus, spina bifida, and myelomeningocele. In Chiari malformation type I, the cerebellar tonsils descend into or beyond the foramen magnum. This form usually has no clinical signs in humans and is often a spurious finding in adults. A 5-mm displacement of the cerebellar tonsils can usually be detected using magnetic resonance imaging (MRI) [13], a safe, non-invasive, and indispensable method for diagnosing Chiari-like malformation. Magnetic resonance imaging is also useful for assessing disease progression and formulating therapeutic options. Chiari malformation type II (also called Arnold-Chiari malformation) in calves and humans is characterised by protrusion (herniation) of the vermis cerebelli into or through the foramen magnum into the spinal canal [14]. It is typically diagnosed in neonates in case of severe neurological signs. A genetic cause is suspected for both malformations, although the trigger has not yet been clarified. In a study of 13 Holstein calves with congenital syndromic Chiari-like malformation, Jacinto, et al. [10] identified various genes with damaged variants, particularly *SHC4* in one case and *WDR45B* in another. One *DYNC1H1* frameshift variant was found to be a candidate causal dominant acting allele in one other case. However, no significant association with the lesion has been established. A common cause for central nervous system (CNS) malformation in calves is *in utero* infection with bovine viral diarrhea virus (BVDV), blue tongue virus (BTV) or Schmallenberg virus (SBV). These viruses can lead to foetal brain malformations when the foetus is exposed in the period when the CNS is vulnerable for viral damage [15–17]. The present case report describes the diagnostic work-up of spina bifida, diplomyelia, and Chiari-like malformation in an Aberdeen Angus calf. The novelty of this case report lies in its interdisciplinary approach to diagnosis; clinical and neurological examinations, radiography and MRI, gross pathological and histopathological evaluations, and comprehensive analysis of genetic material.

## Case presentation

### Signalment/history

A one-day-old, 35 kg, female black Aberdeen Angus calf was admitted to the Clinic for Cattle, University of Veterinary Medicine, Hannover, because of inability to stand. The dam was reared in Estonia and imported to Germany for superovulation and embryo transfer. Artificial insemination with Aberdeen Angus semen imported from the USA was done in Germany. An experienced veterinarian transferred the embryos to six different crossbreed heifers following the German Cattle Breeders Association protocol [18]; only one embryo transfer resulted in pregnancy. The calf was given 1 L of colostrum from a pool of frozen colostrum, obtained from a dairy farm, in the first 24 h after birth.

This case was examined by clinical signs, neurological tests, blood analysis, radiography and magnetic resonance imaging, and postmortem examination. Additionally, the blood specimen was obtained for cytogenetic analysing and whole genome sequencing.

### Clinical findings

**Fig. 1:**
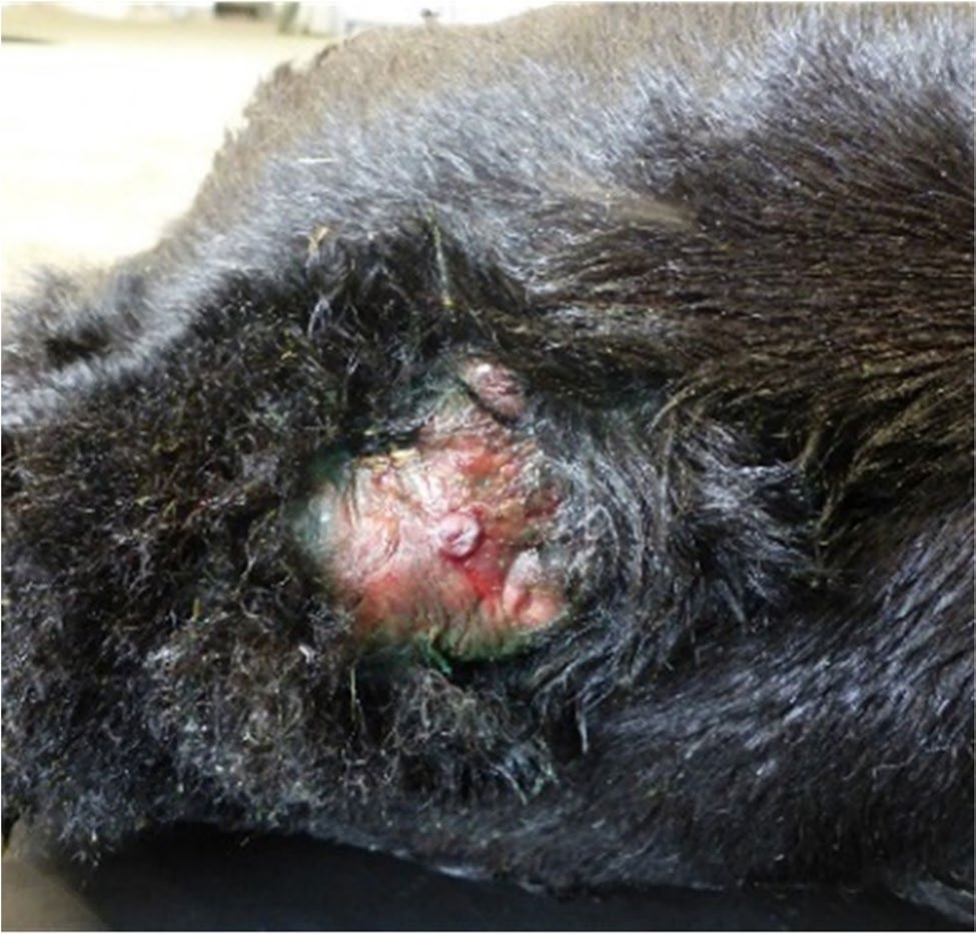
A one-day-old Aberdeen Angus calf with a raised hairless area and epidermal ulceration in the dorsal midline of the sacrum

At admission to the clinic, the calf was alert and in sternal recumbency with the hind limbs extended to one side and the head positioned normally. The calf could not stand even with assistance, or fully extend both carpal joints, although both front limbs could be extended passively. The calf showed motivation to stand up but could only move the front limbs and was not able to rise on its own. A 6 cm wide, circular, pink excrescence with several wrinkles near to its border, which was well demarcated from the normal skin, was present over the caudal sacral region. In the centre of the hairless area, a raised papillary lesion with a central indentation, approximately 0.8 cm in diameter, was visible (Fig. 1). A probe inserted into the fistula-like area could not be extended into the deeper layers. Outflow of exudate or transudate from the excrescence was not observed via the hairless part. The calf was able to suckle milk from a nipple-bottle and remained alert during hospitalisation.

### Neurological examination

Neurological examination revealed normal spinal reflexes in the front limbs. The calf could move the front limbs but could not fully extent both carpal joints. In the hind limbs, paraparesis with decreased muscle tone and severe muscle atrophy was found. The patellar reflex was normal bilaterally, and the flexor reflex response was reduced to absent. The calf remained unable to stand despite daily assistance. The cutaneous trunci reflex was normal bilaterally up to the caudal lumbar area, where no response was elicited. General cutaneous sensitivity was normal in all limbs. Nevertheless, muscle fasciculations were observed in the dorsal skin regions when the hind limbs were stimulated. The tail was flaccid with no active movement. The anal sphincter and tonus were normal. The pupillary light reflex, facial nerve function, palate reflex, and lip tone were normal. The auricular reflex appeared delayed bilaterally; hearing was normal. On the first day postnatum, the calf did not react to visual stimuli. However, in the following days it was noted that visus was not sustained. The animal showed a ventral strabismus in the left eye, ventrolateral strabismus in the right eye, and mild bilateral exophthalmos.

### Treatment

The animal was treated with iron(III)-hydroxide dextran complex (Belfer^®^ 100 mg/ml, Bela-Pharm GmBH & Co. KG, Vechta, Germany, 0.1–0.3 ml/kg BW, s.c.), vitamins A, D, E and selenium (Ursovit^®^AD_3_EC, 30 mg/ml retinyl palmitate, 0.125 mg/ml cholecalciferol, 30 mg/ml alpha-tocopherol acetate, and 100 mg/ml ascorbic acid Serumwerk Bernburg AG, Bernburg, Germany, 5 ml/animal, s.c.), and vitamin E with selenium (Vitamin-E-Selen^®^ ad us vet, 150 mg/ml all-rac-alpha-tocopherol acetate and 1.1 mg/ml sodium selenite, aniMedica GmbH, Senden-Bösensell, Germany, 5 ml/animal supplements).

### Blood analysis

The total leukocyte count (31,900/µl [reference interval 8,000–10,000/µl] [19]), segmented neutrophils (85.0% [22.0–45.0%] [19]), total erythrocyte count (10.4*10^6^/µl [6.0–8.0 *10^6^/µl] [19]), and haematocrit (37.0% [25.0–35.0%] [19]) were increased. Lymphocytes were decreased (13.0% [45.0–65.0%] [19]). Liver variables, including parameters such as total bilirubin concentration (19.6 µmol/l [< 7.0 µmol/l] [19]) and the activity of gamma glutamyl transferase (212 U/l [< 33U/I] [19]) were increased. The concentration of cholesterol (1.43 mmol/l [>3.0 mmol/l] [19]), total protein (49 g/l [60.0–80.0 g/l] [19]) and albumin (25.3 mmol/l [30.0–40.0 mmol/l] [19]) were decreased. A serum ELISA for antibodies against infectious bovine rhinotracheitis virus (IBRV) was negative. Antigen ELISA and serum ELISA testing for BVDV antibodies were negative. Serum ELISA for antibodies was negative for BTV but positive for SBV.

### Radiographic findings

**Fig. 2:**
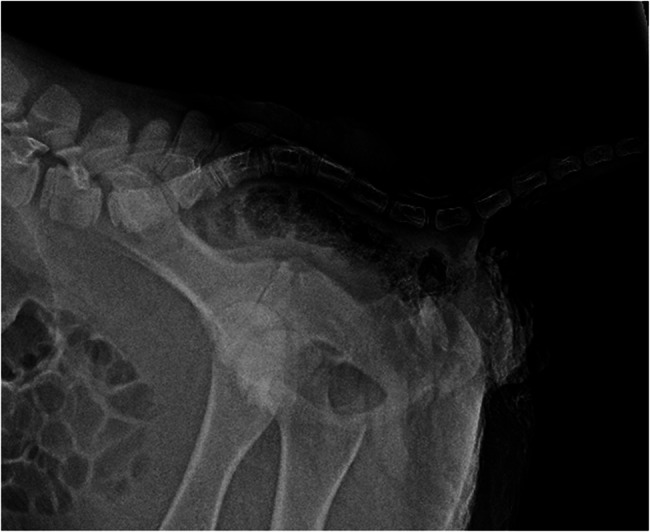
Radiograph showing the S-shaped spinal cord (kyphosis) in a one-day-old Aberdeen Angus calf

Laterolateral and ventrodorsal radiographic of the lumbosacral region were obtained without sedation (Amadeo P-125/100 VB, 5.6 kW, Oehm and Rehbein GmbH Rostock, Germany). The laterolateral view showed that the alignment of the lumbar and sacral spine was dorso-ventrally S-shaped (kyphosis) and absence of the bony dorsal arch in the caudal sacral part of the spinal canal (Fig. 2).

### MRI Findings

**Fig. 3:**
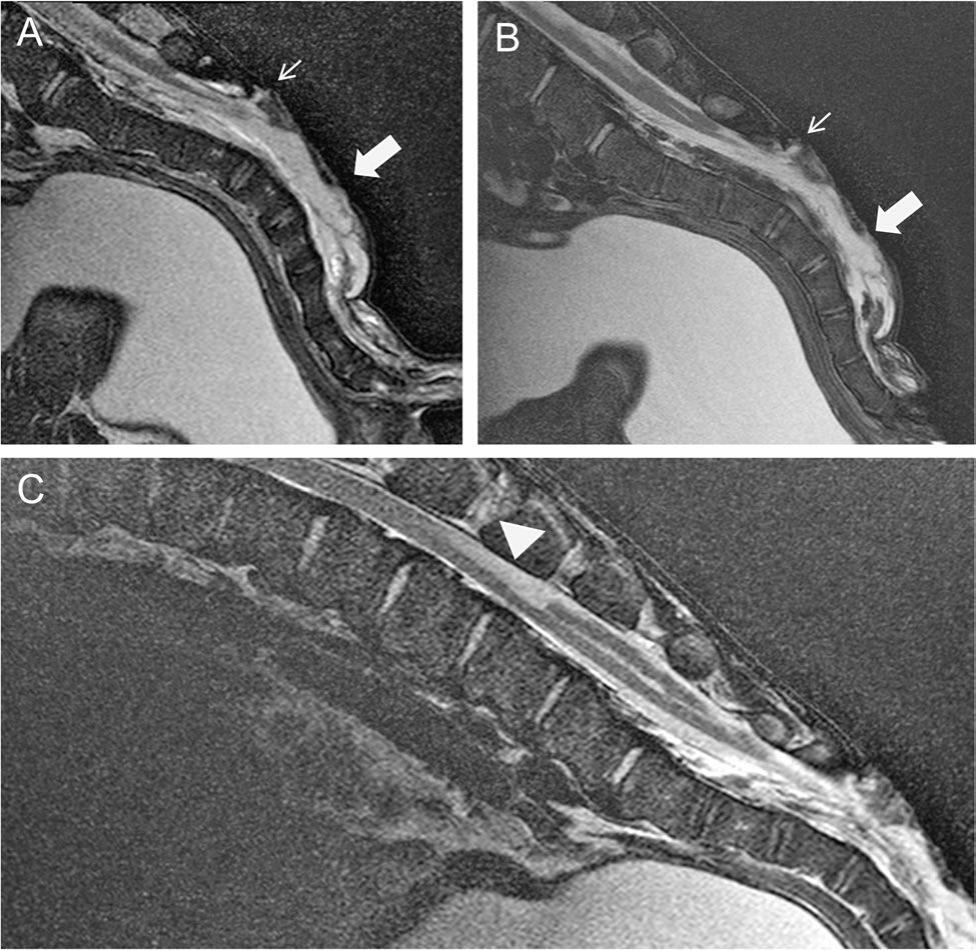
Magnetic resonance imaging of the mid-sagittal lumbosacral area of a 14-day-old Aberdeen Angus calf positioned in lateral recumbency. **A** and **C** T2w images. **B** T2w spectral attenuated inversion recovery. **A + B** show the absence of the dorsal lamina of the sacrum (S3-5). The vertebral canal is filled with hyperintense material (large arrow). The small arrow points to a hyperintense 2 mm wide connection between the dermis and the epidural space. **C** At the level of L4/5, a well demarcated T2w in comparison to the surrounding spinal cord tissue homogeneously hyperintense lesion can be detected in the dorsal region of the myelon (arrowhead). The lesion has the extension of a vertebral body and covers up to one third of the spinal cord tissue

For MRI, general anaesthesia was induced with a combination of 1 ml/100kg xylazine hydrochloride i.m. (Xylavet^®^, 20 mg/ml, CP-Pharma Handelsgesellschaft mbH, Burgdorf, Germany), 0,3 ml/10kg ketamine hydrochloride i.v. (Ketamin^®^, 100 mg/ml, CP-Pharma Handelsgesellschaft mbH, Burgdorf, Germany), and 1 ml/100kg butorphanol i.v. (Butorgesic^®^, 10 mg/ml, CP-Pharma Handelsgesellschaft mbH, Burgdorf, Germany). After administration of ketamine, an intratracheal tube was inserted into the calf’s trachea to maintain anaesthesia with isoflurane (Isofluran^®^CP, 1 ml/ml, CP-Pharma Handelsgesellschaft mbH, Burgdorf, Germany) in oxygen. MRI with a 3 T scanner (Achieva, Philips Medical Systems, Best, The Netherlands) of the lumbosacral area was performed with the calf in lateral recumbency. Pre- and post-contrast T2 weighted (w) sagittal and transverse sequences, T2w sagittal sequences with spectral attenuated inversion recovery (SPAIR), and T1w sagittal and transverse sequences were obtained. In addition, the head was scanned with the calf in dorsal recumbency using a T2w sequence for sagittal and transverse sequences of the brain up to the 2nd cervical vertebra. MRI of the lumbosacral area revealed the absence of the dorsal lamina of S3 to S5. The spinal canal was filled with hyperintense material, similar to the intensity of cerebrospinal fluid and moderately segmented by membranous structures, in T2w and T2w SPAIR sequences. A hyperintense (T2w, T1w), 2 mm wide connection between the dermis and the epidural space was seen cranial to the lesion at S3. A well-delineated, cranially-anchored, homogeneous, hyperintense lesion was remarkable in the dorsal spinal cord at the level of lumbar vertebra (L) 4 and 5 in the T2w sequence (Fig. 3). The dorsal funiculus caudal to this lesion was partially replaced with cerebrospinal fluid up to the sacral area, where it extended to the sacral segmented lesion. The signal intensity of the lumbar spinal cord was normal. Caudal to the fluid accumulation, the central canal was mildly extended to the lumbosacral transition.

**Fig. 4:**
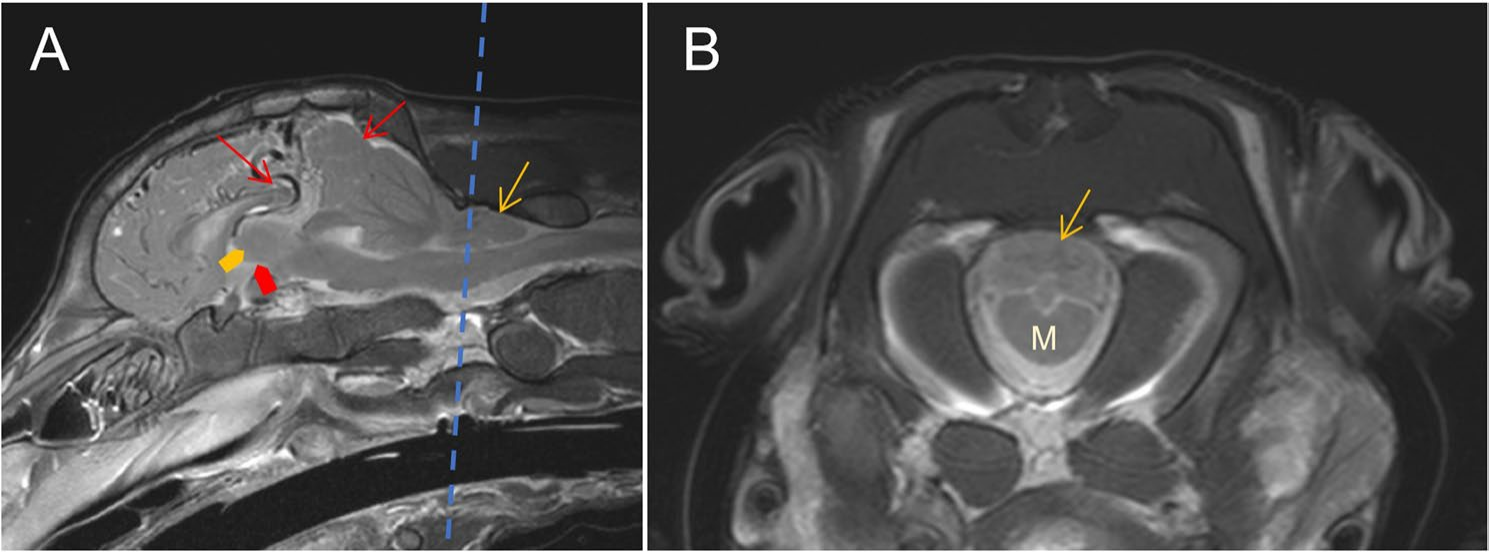
Magnetic resonance imaging of the brain of a 14-day-old Aberdeen Angus calf. The dotted line in (**A**) represents the level of the transverse image in (**B**).** A** T2w mid-sagittal image shows severe herniation of the cerebellum through the foramen magnum (yellow arrow) and subtentorial herniation of the cerebrum (red arrows). The third ventricle (red bold arrows) and the adhesio interthalamica (yellow bold arrow) are misshaped. **B** T2w transverse image at the level of the foramen magnum shows the medulla oblongata (M) with parts of the cerebellum (yellow arrow)

The cerebellar pyramis, uvula, and nodulus were displaced caudally through the foramen magnum 22,63 mm within the spinal canal. The tongue-like structure of the displaced cerebellum penetrated to the cranial quarter of cervical vertebra (C) 1. Caudal subtentorial herniation of the cerebrum was seen. The midsagittal T2w sequence showed a prominent dorsal cerebral sinus and rostral cerebral artery. Diffuse, faintly delineated, hyperintense lesions were seen periventricular to the lateral ventricles and in the brainstem parenchyma in T2w MRI sequences. The latter was at the level of the mesencephalic aqueduct and central canal. The third and fourth ventricles were poorly delineated, appearing as a misshaped interthalamic adhesion (Fig. 4). The cause may have been a secondary ascending inflammation from the S3 connection to the dermis or, more likely, a higher pressure of the cerebrospinal fluid due to the Chiari-like malformation. The intramedullary lesions of the brainstem and ill-delineated interthalamic adhesion are suspected to be due to the cerebellar and subtentorial herniation – as well as the hyperintensity cranial to the cerebellum, most probably caused by a slight dilation of cerebrospinal fluid filled rooms.

### Euthanasia

The owner consented to euthanasia of the calf because of a grave prognosis. Euthanasia was performed immediately after MRI at day 14, with the calf still under general anaesthesia, by intravenous administration of 3 ml/10kg pentobarbital-sodium (Release^®^, 300 mg/ml, WDT, Garbsen, Germany). Euthanasia was conducted according to the method recommended by the Ethics Committee of the Lower Saxony State Office, Veterinary Institute Hannover.

### Postmortem findings

**Fig. 5:**
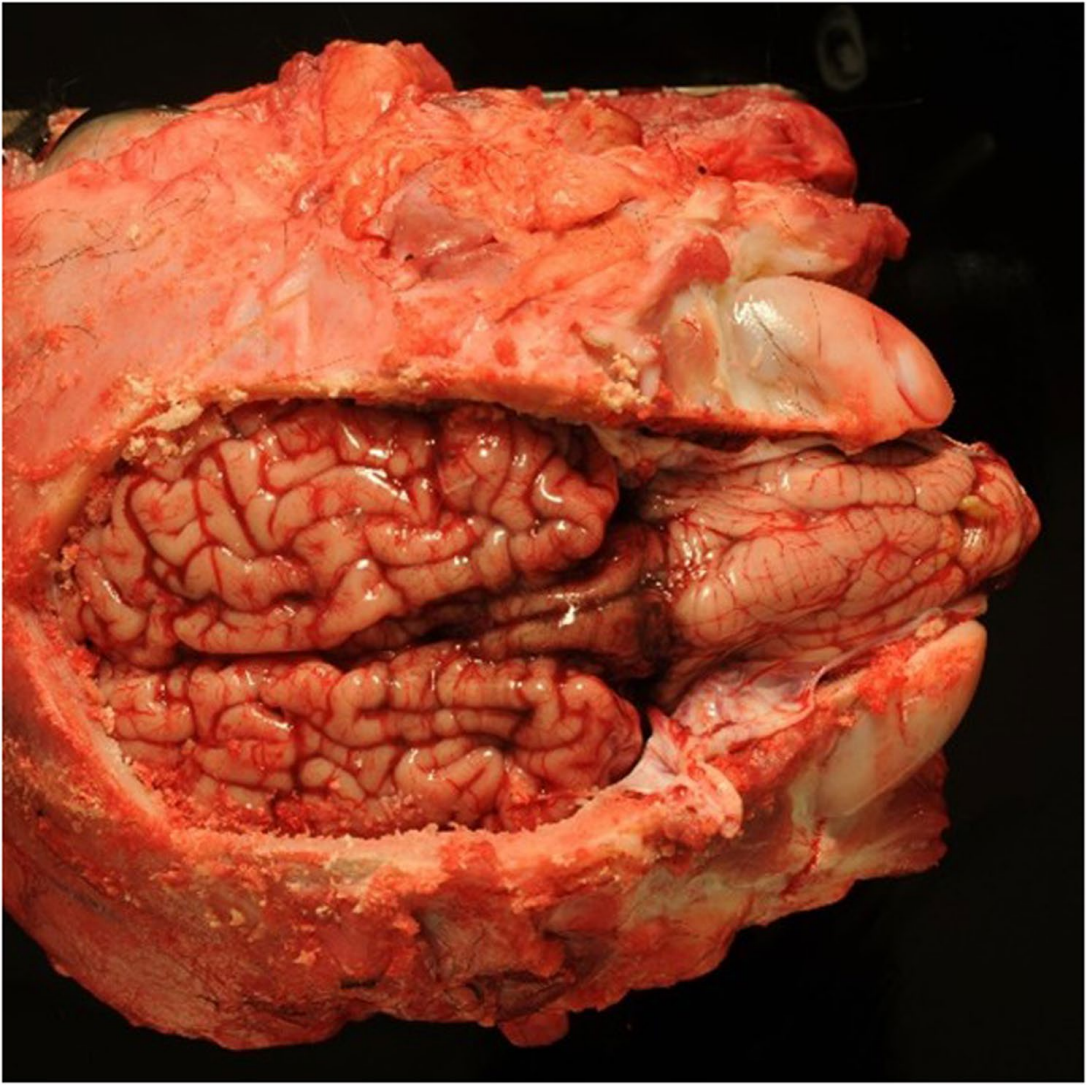
Dorsal view of the brain showing severe herniation, elongation and compression of the cerebellar vermis into the foramen magnum

**Fig. 6:**
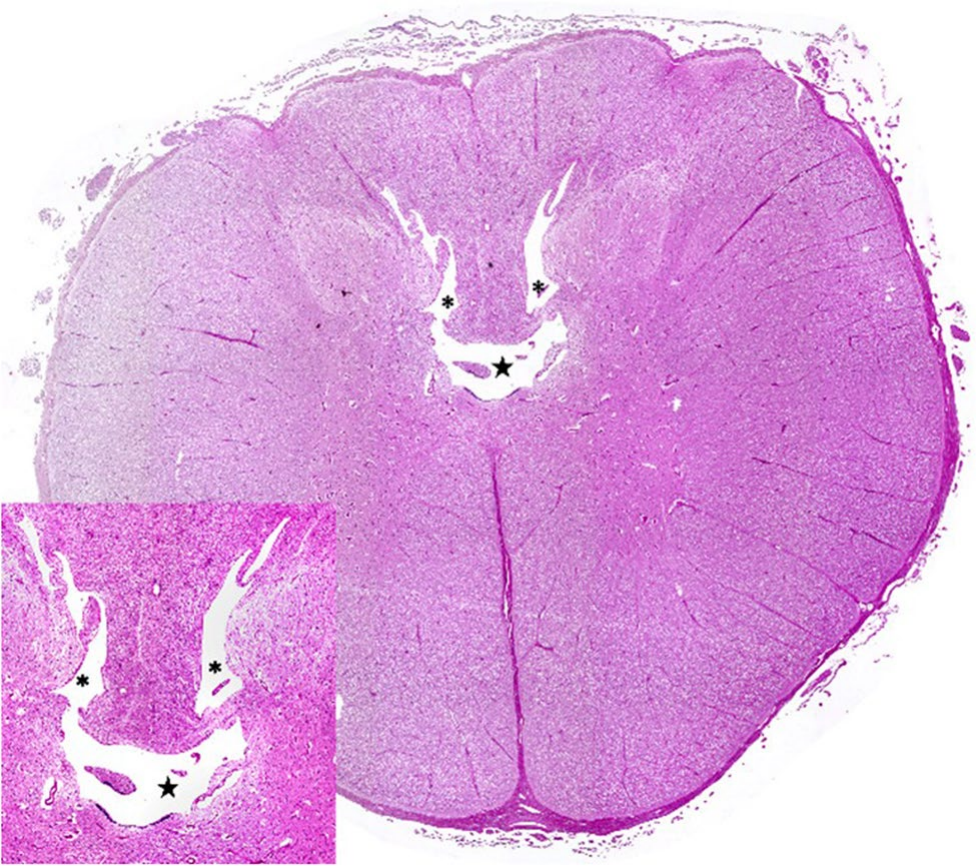
Transverse section of the cervical spinal cord with severe dilatation of the central canal (hydromyelia) and bilateral cavitation of the spinal cord (syringomyelia). The insert shows hydromyelia (★) and syringomyelia (*) in detail. Haematoxylin and eosin stain

**Fig. 7:**
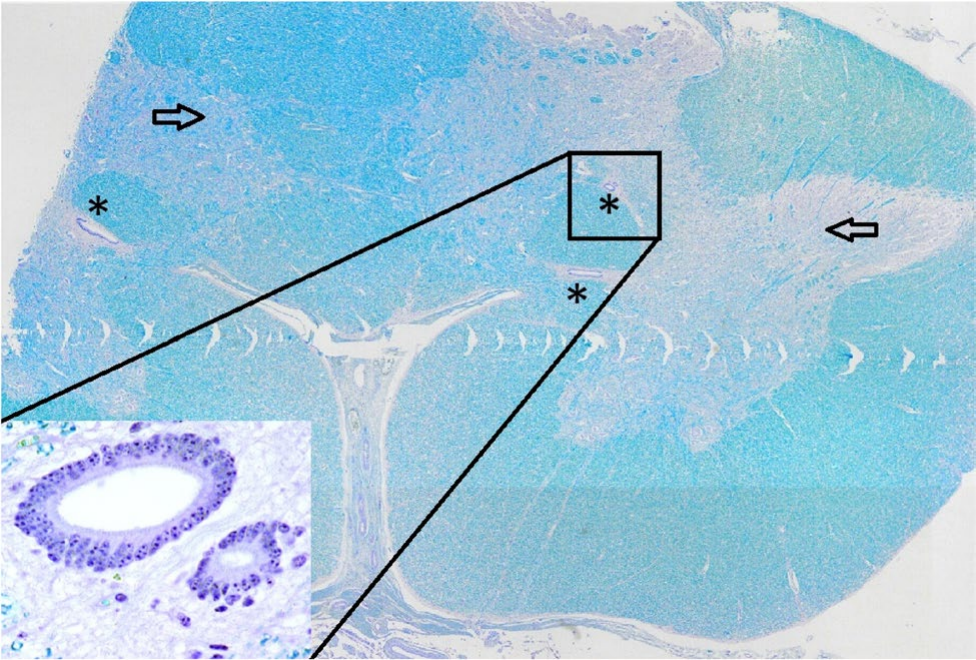
Transverse section of the sacral spinal cord showing duplication of the grey matter (arrow) and multiple central canals (*). The insert is a higher magnification of the central canals. Luxol fast blue stain

Postmortem examination showed severe herniation of the cerebellar vermis into the foramen magnum with associated marked congestion (Fig. 5). The vertebral arches of S3, S4, and S5 were incompletely developed. A 6-cm, circular, hairless area was present at the S3 to S5 midline. Tissue samples were fixed in 10% neutral buffered formalin, embedded in paraffin wax, sectioned, and stained with haematoxylin and eosin (H&E) and luxol fast blue stains. Histological examination of the cervical spinal cord revealed dilatation of the central canal (hydromyelia) and bilateral cavitation of the spinal cord (syringomyelia) (Fig. 6). Transverse sections of the sacral spinal cord showed duplication of the grey matter and central canals (diplomyelia). Additional central canals with a smaller diameter were seen, the smallest measuring 50 μm. Enlargement of the ventral median fissure was present at this location (Fig. 7). The alopecia at the sacral midline area (S3–S5) was characterised by diffuse epidermal ulceration with mild neutrophilic infiltration and granulation tissue formation. In the underlying tissue, mild infiltration of neutrophils, macrophages, and lymphocytes was observed in the arachnoid membrane. Moderate epidermal hyperplasia and mild orthokeratotic hyperkeratosis were seen adjacent to the area of alopecia. Severe suppurative myositis was seen ventral to the area of alopecia. A RT-PCR assay for SBV RNA of spleen and brain tissue carried out by the Lower Saxony State Office, Veterinary Institute Hannover, was negative.

### Cytogenetic analysis

Cytogenetic analyses were performed to identify chromosomal aberrations. The chromosome preparation followed standard protocols using heparinised blood samples for lymphocyte culture preparation [20]. The metaphase chromosomes were stained with Giemsa and examined with a light microscope (Axioplan 2, Carl Zeiss Microscopy, Jena, Germany). Photos were taken with a computer-controlled CCD camera (Axiocam 105 color, Carl Zeiss Microscopy) and processed with ZEN 2 (blue edition) software (Carl Zeiss Microscopy). A total of 50 metaphase chromosomes were analysed, all displaying a normal karyotype with 2n = 60, XX chromosomes. Numerical or chromosomal aberrations of autosomal and sex chromosomes were not observed in any of the metaphase chromosomes.

### Whole genome sequencing

For whole genome sequencing (WGS), genomic DNA was isolated from an EDTA-blood sample of the affected calf and semen samples of the sire of the affected calf using standard chloroform extraction. According to the manufacturer’s protocols libraries from these samples were prepared using randomly fragmentation of 1µg genomic DNA. Thereafter, selection by Magnetic beads to an average size of 200–400 bp were done. Fragments were end repaired and then 3’ adenylated. Adaptors were ligated to the ends of these 3’ adenylated fragments. PCR followed to amplify fragments with adaptors from previous step. PCR products were purified by the Magnetic beads. Denaturation and circulation following by the splint oligo sequence. Size selection and indexing was quality controlled on an Agilent 2100 Bioanalyzer system with a High Sensitivity DNA kit (Agilent Technologies, Santa Clara, CA). WGS was performed using a DNBSEQ-T7 (formerly known as MGISEQ-T7) in a 2 × 150 bp paired-end mode. Quality control for the data was performed using fastqc 0.11.9 [21] and the reads were trimmed using SOAPnuke software (-n 0.001 -l 10 -q 0.4 -A0.25 -Q 2 -G --cutAdaptor --minLen 150) (BGI, HongKong, China). The resulting data were mapped to the bovine reference genome ARS-UCD1.2 (Ensembl) using BWA 0.7.17 [22] resulting in a mean coverage of 19.5X for the case (average mapping rate 99.5%) and 19.7X for its sire (average mapping rate 99.4%) and 10-19X for the controls. SAMtools 1.11 [23] and Picard tools (http://broadinstitute.github.io/picard/, version 2.25.0) were used for sorting, indexing and marking of duplicates of bam-files. Variants were called with GATK, version 4.2.0.0 [24], using Base Quality Score Recalibration (BQSR), Haplotype Caller and Variant Recalibrator. The variants of the two sequenced animals were compared with variant callings from 34 private controls including cattle of the breeds Holstein, Fleckvieh, Braunvieh, Vorderwald, German Angus, Galloway, Limousin, Charolais, Hereford, Tyrolean Grey and Miniature Zebu. All variants with high or moderate effects according to prediction toolbox SNPEff, version 5.0 e (2017-08−30, SNPEff database ARS-UCD1.2_release 103) [25] were selected. The WGS data of the affected calf and its sire were screened for mutant variants using SAS, version 9.4 (Statistical Analysis System, Cary, NC, USA). We filtered out variants homozygous for the affected calf and heterozygous for the sire for the case of a recessive inheritance, or heterozygous for the affected calf but not shared by the sire for the case of a spontaneous mutation or germline mutation. Predicted effects of these private variants were investigated using the Variant Effect Predictor [26] for SIFT [27] predictions and PolyPhen-2 [28]. All variants were searched for candidate genes based on the literature. A keyword search for publications related to spina bifida in humans or domestic animals and based on the clinical signs observed in the affected calf with searching of the following words ‘cerebellar displacement’, ‘arnold-chiari malformation’, ‘type II chiari malformation’, ‘arnold-chiari-like’, ‘dysraphic disorder’, ‘spinal dysraphism’, ‘occipital encephalocels’, ‘chiari-like malformation’, ‘caudal occipital malformation’, ‘occipital hypoplasia’, ‘hydrocephalus’, ‘internal hydrocephalus’ or ‘meningomyelocele’ to detect genes associated with such phenotypes was conducted using NCBI gene (https://www.ncbi.nlm.nih.gov/gene) and Ensemble (https://www.ensembl.org/index.html). The purpose was to find variants in the calf but absent in the sire or variants in the calf and the sire but absent in 34 control samples from 11 different breeds. Reads were mapped to the ARS-UCD1.2 bovine reference genome, resulting in a mean coverage of 19.5X for the calf, 19.7X for the sire, and 10-19X for the 34 controls. The calf, sire, and 34 control samples had 34.288.637 single base pair variants and 5.513.879 indels. The calf had 40.138 variants affecting protein function (additional file 1), and the calf and sire had 27.496 (additional file 2). Homozygous mutated variants in the calf and heterozygous variants in the sire yielded 1588 results (additional file 3). A total of 46 variants remained after filtering for variants with high or moderate effects and variants associated with genes related to the following diseases (additional file 4): cerebellar displacement, Arnold-Chiari malformation, type II Chiari malformation, Arnold-Chiari-like malformation, dysraphic disorder, spinal dysraphism, occipital encephalocele, Chiari-like malformation, caudal occipital malformation, occipital hypoplasia, hydrocephalus, internal hydrocephalus, and meningomyelocele (additional file 5). This procedure was done according to SNPEff predictions on protein structure. The intronic splice acceptor variant (g.19087428 C >CTTGATAT, c.879-1_879insATATCAA) within *GRM7* on BTA22 (ARS-UCD1.2) was the most likely candidate due to its protein impact through altered splicing.

## Discussion

The history and clinical examination in the present case ruled out metabolic, infectious, and traumatic causes of inability to stand. Serum ELISA was positive for SBV antibodies, but SBV RNA was not found in spleen and brain samples. Passive transfer of colostral SBV antibodies may explain the presence of antibodies in the calf. Radiography and MRI showed the presence of several congenital anomalies, and postmortem examination confirmed spina bifida, diplomyelia, and Chiari-like malformation. These anomalies have been often described together in cattle and humans [3, 29]. Chiari malformation type I is the most common form in humans, followed by type II; however, determining the prevalence is difficult because many people with type I are symptom-free and the malformation is usually an incidental finding [13]. A hereditary cause of type I with abnormal regions on chromosomes 1 and 22 was proposed [30]. Chiari type I malformation in dogs is usually restricted to certain breeds, particularly the Cavalier King Charles Spaniel. The disease often becomes apparent when adult dogs exhibit compulsive behaviours. A recessive mode of inheritance was determined [31, 32]. In the present case, the cerebellar tonsil was 22,63 mm dislocated through the foramen magnum. In combination with the severe deformation of the cerebellum, this results in a high degree of malformation. A similarly severe case of cerebellar herniation through the foramen magnum and dislocation of the brainstem was described by Sato, et al. [33] with a protrusion of 12.78 mm and syringomyelia in the C2-3 region as visualized by MRI. In contrast to our present case, that calf was able to stand with difficulty on the 5th day of life. The Chiari-like malformation was classified as type 1.5, and a better prognosis was assumed for this form. In the present case, there were other major lesions, such as spina bifida and diplomyelia, which further worsened the prognosis. Spina bifida occulta means that the vertebral bones are not fully closed around the spinal cord during development, but this is not visible externally as the lesion is covered by skin [8]. In the present case, the spinal cord was covered with skin over the whole region. Spina bifida usually develops over several vertebrae but in this case only at the level of S3 there was a tubular structure right under the skin surface which communicated with the subarachnoidal space. Other authors have described spina bifida in calves with exposed spinal cord [3, 34, 35].

In the present case, the spinal reflexes of the front limbs were normal, but the calf was unable to stand on its front limbs or fully extend the legs. This could be due to constriction of the brain stem and medulla oblongata caused by herniation of the cerebrum and protrusion of the cerebellum. The withdrawal reflex primarily tests sciatic nerve motor function, while hip flexion is mainly induced by femoral nerve innervation. Inability to stand on the hind limbs may have been attributable to altered cerebellar anatomy, as the caudal vermis coordinates motor function of the lower trunk and pelvic region. Compression of the caudal cerebellar hemispheres may further impact pelvic limb function, explaining the observed motor deficits in our case. Reduced hip flexion may have been due to a suspected subarachnoid fluid accumulation at L4-5, combined by a still present patellar reflex. The anatomical alterations in the sacral region and fluid accumulation may have also affected sciatic nerve function. Syringomyelia and cerebellar tonsillar herniation have been described in calves as causes of hind limb paralysis and lack of pelvic limb withdrawal reflexes [2, 33]. Górriz-Martín, et al. [36] reported that a split spinal cord malformation in the lumbosacral segments results in hind limb neurological deficits. Diplomyelia in the cervical or thoracic segments can lead to the absence of superficial and deep sensitivity responses in the thoracic limbs [37]. A crossbreed calf with focal diplomyelia in the L4 area had typical ‘bunny hopping’ locomotion [38]. The withdrawal and patellar reflex responses in the hind limbs were normal but decreased and eventually became absent within four weeks; deterioration of the reflex responses was thought to be caused by the lesion and weight gain. Muscle atrophy is frequently observed depending on diplomyelia and syringomyelia. To the authors’ knowledge, exophthalmos and strabismus were not described in other case reports of calves with similar lesions. This could be because in some cases the calves were stillborn, and pathological eye conditions were not investigated. Exophthalmos and ventral strabismus have been described as accompanying signs of Chiari malformation in the Cavalier King Charles Spaniel [39], however, the eyeball malposition was attributed to brachycephaly. The strabismus in the present case may have been due to an oculomotor nerve (cranial nerve III) lesion, possibly related to the midbrain lesion and caudal subtentorial herniation. Impaired vision may also have been associated with midbrain compression and abnormal occipital cortex anatomy. Brain herniation describes the displacement of brain structures from their normal position within the skull [40]. The tentorium cerebelli is a firm layer of the dura mater that divides the cranium into the supratentorial compartment with the cerebral hemispheres and the infratentorial compartment with the cerebellum and brain stem [41]. In the present case, subtentorial herniation of the brain refers to shifting of cerebral structure under the tentorium by distorting cerebellar structures. dos Santos, et al. [42] described caudal extent of the occipital lobes “underneath the tentorium cerebelli” in a calf with similar neural tube and skeletal malformations. The gross pathological examination revealed a V-shaped deformation of the occipital lobes. However, MRI was not performed in the living animal. LeClerc, et al. [4] and Jacinto, et al. [10] also observed caudal elongation of the occipital lobes in five similar cases. The affected animals were all described as having flattened neurocrania, which was not a finding in the present case. In other animal species and humans, transtentorial herniations through the tentorial notch have been described. Trauma and increased intracranial pressure are often the causes of cerebral displacement [43]. Tetraplegia, coma and apnoea were common signs of caudal transtentorial herniation in dogs, cats and a horse. Pupil dilation, paraplegia and respiratory depression were observed in humans with this lesion. Lewis, et al. [44] developed a morphometry to scale the severity of brain herniation in dogs and cats. They did not associate any specific neurological signs with caudal transtentorial herniation. Trauma, neoplasia and increased intracranial pressure were cited as common causes in these species. However, clinical examination and diagnosis of spinal cord and brain malformations can be difficult because of the overlapping of deficiencies and signs caused by different lesions. The location, type and extent of the lesion also affect clinical signs.

In all animal species, imaging techniques are required for diagnosing spina bifida and other associated congenital anomalies because clinical signs might be nonspecific. Diagnosis of caudal brain disorders using ultrasonography has been described in calves and dogs [2, 45]. However, Chiari-like malformation in humans and dogs is commonly diagnosed with MRI [45, 46]. MRI is an important, non-invasive diagnostic tool, providing detailed images of the CNS and other soft tissues. Clinical and neurological examinations can effectively determine the localisation of CNS lesions or malformations, but the actual cause can only be identified through imaging techniques. MRI allows precise assessment of these conditions and enhancing our understanding of the pathophysiology. Surgical intervention consisting of decompression and drainage is possible in humans and dogs [46, 47]. However, surgical treatment in calves is not done because the effort outweighs the economic benefit. Especially in connection with the other malformations, it seems appropriate from an animal welfare point of view to euthanise affected calves with severe clinical signs.

Whole genome sequencing revealed metabotropic glutamate receptor 7 (*GRM7*) as a candidate locus for the congenital malformations in the present case. *GRM7* has not yet been associated with malformations such as those described in the present case, but other reports described an effect of *GRM7* on brain development during neurogenesis [48]. In humans, *GRM7* is associated with developmental brain defects, such as attention deficit hyperactivity disorder and autism [49, 50]. In dogs, *GRM7* is associated with fearfulness and noise sensitivity [51]. To the authors’ knowledge, a link between *GRM7* and neurological disorders in cattle has not been established. The reason may be that few studies focus on behavioural abnormalities in cattle, and research on livestock behaviour is sparse. Only up-regulation of *GRM7* during superovulation *in utero* has been described in cows [52]. Other genomic defects have been described, including Chiari-like malformation in Holstein calves. However, no significant association with any gene has been established [10].

## Conclusion

Although congenital malformations occur relatively rarely in cattle, pursuing a definitive diagnosis is important in affected calves, particularly for breeding reasons.

## Supplementary Information

Supplementary Material 1.

Supplementary Material 2.

Supplementary Material 3.

Supplementary Material 4.

Supplementary Material 5.

## Data Availability

All data generated or analysed during this study are included in this published article and its supplementary information files.

## Abbreviations

BTV: Blue tongue virus
BVDV: Bovine viral diarrhea virus
ELISA: Enzyme-linked Immunosorbent Assay
C: Cervical vertebra
CNS: Central nervous system
GRM7: Metabotropic glutamate receptor 7
i.m.: Intramuscular
i.v.: Intravenous
L: Lumbar vertebra
MRI: Magnetic resonance imaging
RT-PCR: Reverse transcription polymerase chain reaction
S: Sacral vertebra
SBV: Schmallenberg virus
SPAIR: Spectral attenuated inversion recovery
WGS: Whole genome sequencing
